# Bacillary angiomatosis presenting with facial tumor and multiple abscesses: A case report

**DOI:** 10.1097/MD.0000000000004155

**Published:** 2016-07-18

**Authors:** Mateusz Markowicz, Stephanie Käser, Andreas Müller, Gerold Lang, Susanna Lang, Marius Mayerhöfer, Gerold Stanek, Armin Rieger

**Affiliations:** a Institute for Hygiene and Applied Immunology, Center for Pathophysiology, Infectiology and Immunology; b Division of Immunology, Allergy and Infectious Diseases, Department of Dermatology; c Clinical Institute for Pathology; d Department of Radiology and Nuclear Medicine, Medical University of Vienna, Vienna, Austria.

**Keywords:** abscess, bacillary angiomatosis, *Bartonella quintana*, case report, culture, HIV, PCR

## Abstract

**Background:**

The clinical manifestation of bacillary angiomatosis (BA) can be limited to one organ, most commonly the skin, but systemic courses can also occur. We report a human immunodeficiency virus (HIV)-positive patient with a systemic manifestation of BA caused by *Bartonella quintana*, diagnosed in Vienna, Austria. The pathogen was detected in multiple organs including a facial tumor which is an unusual finding for BA. Furthermore, infections with *B quintana* are rare in our area and no other autochthonous cases have been reported.

**Methods and results:**

The clinical manifestation included multiple papules and nodules on the entire body, several organic abscesses, and a facial tumor influencing the patient's view.

The main laboratory finding indicated HIV infection combined with severe immunosuppression with 47 CD4^+^ cells/μL. Contrast-enhanced computed tomography of the chest and the abdomen showed multiple and abscesses. Histological examination of the facial tumor confirmed inflammatory process. *Bartonella quintana* was detected by PCR in blood and in the facial tumor as well as by culture in the skin tissue. Antibiotic treatment with doxycycline and antiretroviral therapy resulted in clinical improvement.

**Conclusion:**

Our case shows that rare opportunistic, vector-borne infections, usually associated with poverty, can lead to diagnosis of HIV even in well-developed countries. Furthermore, we provide details on clinical manifestation and diagnostic work-up which might expand the knowledge on disseminated infections with *B quintana*. As far, tumorous deformations have rarely been reported as consequence of BA. In our patient the pathogen was detected in the facial tumor using PCR techniques.

## 1 Introduction

Bacillary angiomatosis (BA) is an angioproliferative disease caused by *Bartonella henselae* and *Bartonella quintana*. It predominantly affects the skin of immunocompromised, particularly human immunodeficiency virus (HIV)-positive patients. Since the first isolation of the pathogen from human tissue,^[1]^ reports on clinical manifestations rapidly expanded in both immunocompromised and immunocompetent patients. *B henselae* has a zoonotic reservoir and can be acquired by contact with cats.^[2]^ Humans are the main reservoir of *B quintana* and it is transmitted by the louse *Pediculus humanus* but it was also found in fleas.^[3]^ This is a report on a disseminated infection with *B quintana* in an HIV / hepatitis C virus (HCV)-coinfected patient diagnosed in Austria. Clinical manifestations of BA included constitutional symptoms, typical skin manifestations, multiple abscesses of abdominal and thoracic organs, and a tumorous deformation of the face. A signed consent form was obtained from the patient.

## 2 Case presentation

**Figure 1:**
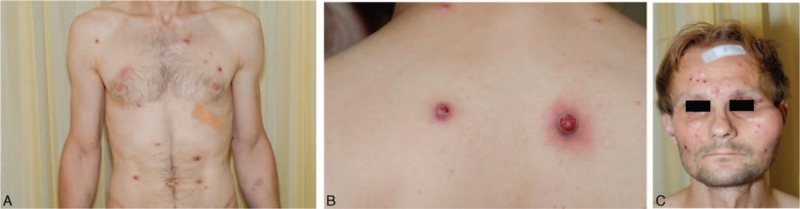
Clinical presentation of the patient with bacillary angiomatosis. (A) Multiple red papules on the chest and abdomen. (B) Magnification of 2 nodules on the back. (C) Papules on the face with a mass lesion at the left zygomatic arch.

In September 2015, a 36-year-old, emaciated, Hungarian man presented with a 3-month history of widespread, red, rust-colored papules and nodules partially erosive and crusted as well as a 4 cm wide, prominent, skin-colored, subcutaneous tumor at the left zygomatic arch (Fig. 1). The oral mucosa was free of lesions. The patient was occasionally homeless in Austria, his travel history was unremarkable and he reported intravenous drug use. The latter comorbidity and the clinical presentation prompted HIV rapid testing, which provided a positive result.

HIV infection was confirmed by Western blot and quantitative HIV PCR (4.70 log copies/mL). The CD4^+^ cell count was 47 cells/μL (CD4^+^/CD8^+^ ratio: 0.06), indicative for severe immunosuppression. In addition, the patient was HCV positive and the viral load was 2.62 ×10^6^ copies/mL (genotype 1a). The white blood cell count was within normal range, and the patient was subfebrile.

Related to the rust-colored papules, BA, vascular tumors-like multiple pyogenic granulomata (PG), and HIV-associated Kaposi sarcoma were suspected. A biopsy of the nodular skin lesions from the dorsum revealed solid, abundant telangiectatic vessels in the upper dermis with prominent endothelial cells surrounded by neutrophils and lymphocytes, compatible with BA and PG. Immunohistochemical staining for human herpes virus 8 was negative. The Warthin–Starry stain showed clusters of microorganisms. High levels of serum IgG antibodies against *B henselae* and *B quintana* were detected in an indirect immunofluorescence assay (Euroimmun, Lübeck, Germany) strongly suggesting BA.

For direct pathogen identification, an EDTA blood sample and tissue samples of the nodular skin lesions were analyzed in PCR assays. DNA was extracted and processed from both samples using a peqGOLD Tissue DNA Mini Kit (Peqlab) according to the manufacturer's protocol. DNA isolates were amplified with a real-time PCR targeting the *ssrA* gene of *Bartonella* spp.^[4]^ An ABI7900 cycler (Applied Biosystems, Foster City, CA, USA) was used with a thermal profile of initial enzyme activation at 95°C for 2 minutes, 45 cycles of denaturation at 95°C for 15 seconds, followed by annealing and elongation at 61°C for 1 minute. *Bartonella* spp. DNA was detected in all samples. Amplicons for sequence analysis were generated in conventional PCR. The PCR products were loaded onto a 1% agarose gel and amplicons of size 301 bp were purified using the QIAquick Gel Extraction Kit (Qiagen, Austria). DNA sequencing was carried out by Eurofins Genomics DNA sequencing service (Eurofins Genomics GmbH, Ebersberg, Germany). The forward and reverse DNA sequences obtained were used for homology searches in the National Center for Biotechnology Information (NCBI) database BLAST search program (http://www.ncbi.nlm.nih.gov/blast).

The skin tissue was further cultured on Columbia blood agar plates (Biomerieux, Vienna, Austria) at 37°C in an atmosphere of 5% CO_2_ and 95% humidity. After 4 weeks of culture, microbial growth was detected by clinical characteristics and selected colonies were propagated by culture via dilution streak plating. Single colonies were then picked and sub-cultured, and the DNA was purified and tested by a *Bartonella*-specific PCR assay.^[4]^ Sequencing (BLAST analysis) demonstrated a clearly positive result for *B quintana.*

**Figure 2:**
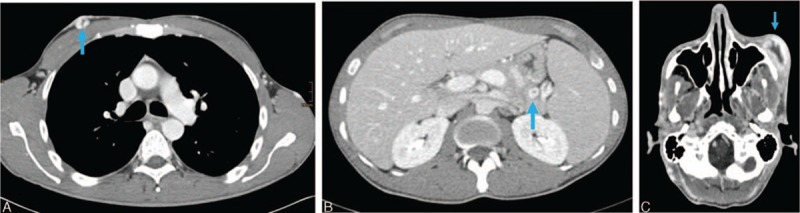
Contrast-enhanced CT of the patient with bacillary angiomatosis showing multiple abscesses (blue arrows). Axial CT images. (A) Chest: subcutaneous abscess in contact with the right pectoralis muscle. (B) Abdomen: abscess in the tail of the pancreas. (C) Head: massive abscess at the left zygomatic arch with bone erosion. CT = computed tomography.

Contrast-enhanced computed tomography (CT) scans of the head, chest, and abdomen (Fig. 2) revealed hepatosplenomegaly and several abscesses located in lung, stomach wall, pancreatic tail, subcutaneous tissue, and the large lesion (4.5 × 2.8 cm) at left zygomatic arch already eroding the adjacent bone. No pathological findings were seen on x-ray of the long bones or on echocardiography.

In order to exclude a different pathology of the facial subcutaneous mass, such as Burkitt lymphoma, a biopsy was performed. Histology revealed neutrophil infiltrations with no evidence for a malignant process. In addition, DNA was extracted from the paraffin-embedded tissue for broad spectrum fungal PCR (MycoRealFungi, Ingenetix, Vienna, Austria) and no pathogens could be identified. The same tissue was tested for *B quintana* by PCR as described above and the result was positive. Silver impregnation staining showed bacilli.

In view of the advanced immunosuppression, a screening for other opportunistic infections was performed. Atypical and typical mycobacterial infections were excluded by negative cultures from blood, urine, and skin biopsies. The TBC-specific interferon-gamma release assay and *Cryptococcus neoformans* antigen were also negative. Among several blood cultures from venous blood only one was positive for *Staphylococcus epidermidis.*

After 23 days of treatment with doxycycline 100 mg bid, an improvement of the skin lesions, partial regression of the facial tumor and resolution of fever were observed. The HIV viral load had decreased to 2.02 log c/mL after initiation of antiretroviral therapy with emtricitabine/tenofovir 200/245 mg qd and dolutegravir 50 mg qd. A follow-up CT scan could not be performed, because the patient abandoned the hospital and was lost for follow-up.

## 3 Discussion

The reported case of culture confirmed BA stands out among other known manifestations of the disease because of several reasons. First, the pathogen was identified by PCR and silver staining in a large subcutaneous abscess which is a very rare manifestation of BA, and only single, similar cases have been reported.^[5]^ Second, the histopathological examination of the facial tissue showed no proliferation of blood vessels typical for BA but it was rather compatible with an inflammatory process. Contrast-enhanced CT scans showed similar lesions at intrathoracic and intraabdominal locations. Although the pathogenesis of the abscesses has not clearly been demonstrated, series of blood cultures gave no convincing evidence for another infectious agent involved in the disseminated infection. Therefore, circumstantial evidence exists that all other abscesses were caused by *B quintana* as well.

In order to compare the clinical manifestation of BA in our patient to other known cases we performed a review of the literature with special focus on patients with typical skin manifestations and concomitant organ involvement. The main inclusion criterion was isolation of *Bartonella* spp. by culture or PCR and affection of several organs. The results are demonstrated in Table 1. Based on this review, we conclude that our patient presented with an extensive systemic course of the disease which was rarely observed previously. Particularly, no other patient presented with a facial mass and no isolation of *B quintana* from such lesion has been reported.

**Table 1:**
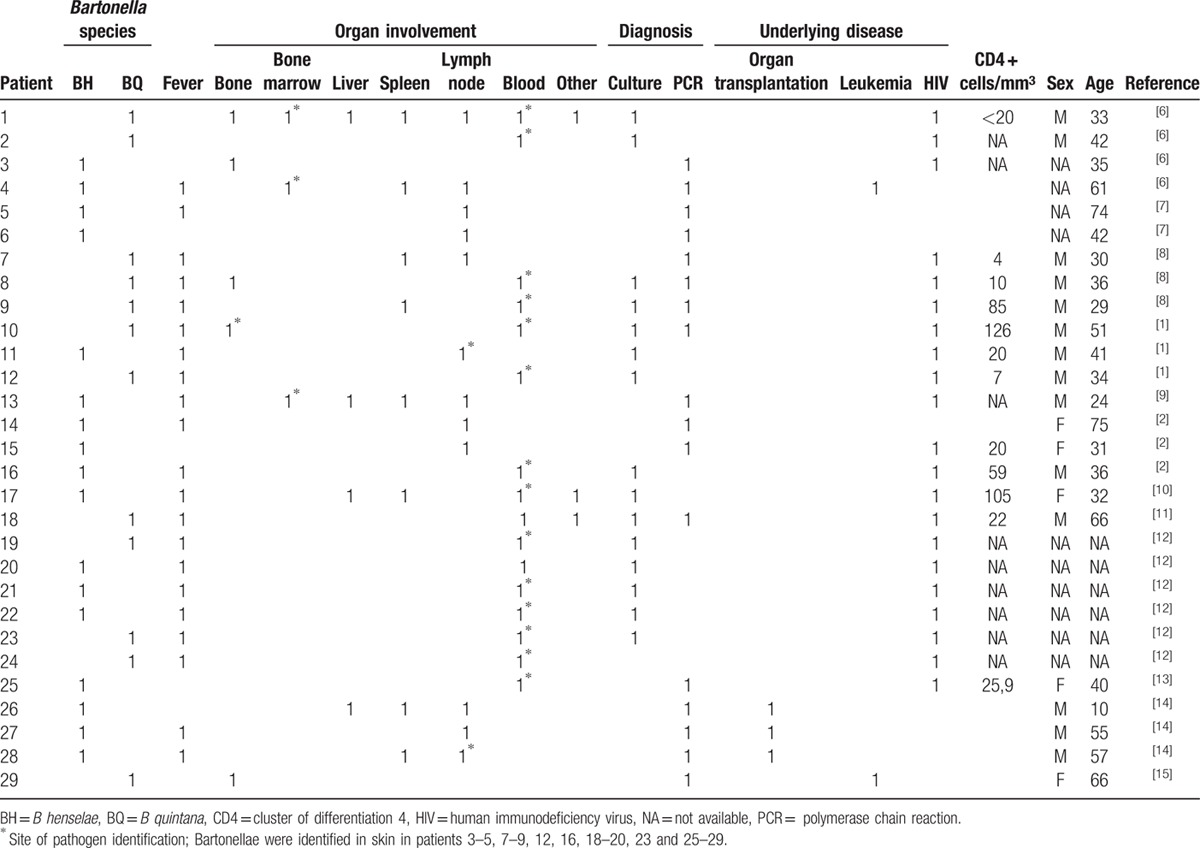
Review of published cases of bacillary angiomatosis with multiple organ involvement. Pathogen was identified using PCR or culture.

Successful management of Bartonella infections in HIV-positive patients is based on antimicrobial therapy combined with antiretroviral treatment to reverse HIV-associated immunosuppression. In our patient, the treatment proved to be effective after nearly 3 weeks. However, no further follow-up was possible to assess its long-term effect.

Our report should raise awareness that even in economically developed countries, late presentation with HIV infection with or without opportunistic infection is an ongoing challenge. In our patient, occurrence of a rare, vector-borne disease usually associated with poverty and bad hygienic standards leads to diagnosis of HIV. Noteworthy, under certain conditions like homelessness, poverty, chronic alcohol abuse, and poor hygienic standards associated with lice infestation, *B quintana* can also affect immunocompetent individuals causing trench fever, endocarditis, and chronic bacteremia.^[3]^ In this respect, more epidemiological data are needed to assess the risk for acquiring *B quintana* and to identify potential sources of infection in our area.

## Acknowledgments

The authors thank Georg Stingl, Hannes Stockinger, Harald Kittler, and Birgit Willinger for their valuable comments and helpful discussions on this manuscript. Permission to be named was obtained.
